# Investigations of Differential Hypoxemia During Venoarterial Membrane Oxygenation with and Without Impella Support

**DOI:** 10.1007/s13239-024-00739-w

**Published:** 2024-06-27

**Authors:** Michael Neidlin, Ali Amiri, Kristin Hugenroth, Ulrich Steinseifer

**Affiliations:** 1 Department of Cardiovascular Engineering, Institute of Applied Medical Engineering, Medical Faculty, RWTH Aachen University, Forckenbeckstr. 55, 52074 Aachen, Germany; 2 enmodes GmbH, Aachen, Germany

**Keywords:** Computational fluid dynamics, Extracorporeal membrane oxygenation, Oxygen transport, Mechanical circulatory support, Impella, left ventricular unloading

## Abstract

**Purpose:**

Venoarterial extracorporeal membrane oxygenation (VA ECMO) is used in patients with refractory cardiac or cardio-pulmonary failure. Native ventricular output interacts with VA ECMO flow and may hinder sufficient oxygenation to the heart and the brain. Further on, VA ECMO leads to afterload increase requiring ventricular unloading. The aim of the study was to investigate aortic blood flow and oxygenation for various ECMO settings and cannula positions with a numerical model.

**Methods:**

Four different aortic cannula tip positions (ascending aorta, descending aorta, abdominal aorta, and iliac artery) were included in a model of a human aorta. Three degrees of cardiac dysfunction and VA ECMO support (50%, 75% and 90%) with a total blood flow of 6 l/min were investigated. Additionally, the Impella CP device was implemented under 50% support condition. Blood oxygen saturation at the aortic branches and the pressure acting on the aortic valve were calculated.

**Results:**

A more proximal tip orientation is necessary to increase oxygen supply to the supra-aortic and coronary arteries for 50% and 75% support. During the 90% support scenario, proper oxygenation can be achieved independently of tip position. The use of Impella reduces afterload by 8–17 mmHg and vessel oxygenation is similar to 50% VA ECMO support. Pressure load on the aortic valve increases with more proximal tip position and is decreased during Impella use.

**Conclusions:**

We present a simulation model for the investigation of hemodynamics and blood oxygenation with various mechanical circulatory support systems. Our results underline the intricate and patient-specific relationship between extracorporeal support, cannula tip orientation and oxygenation capacity.

**Supplementary Information:**

The online version contains supplementary material available at 10.1007/s13239-024-00739-w.

## Introduction

Venoarterial extracorporeal membrane oxygenation (VA ECMO) is used in patients with cardiogenic shock or simultaneously impaired lung and heart function. The extracorporeal system supports both the heart and the lungs. Blood is withdrawn from a central vein and returned, retrograde, through an artery, with the aorta commonly chosen for the return in most cases [1]. The return cannula is usually inserted into the femoral artery and advanced to the iliac artery; thus, the blood from the extracorporeal circuit is returned against the native flow direction into the body [2]. Generally, any form of ECMO therapy is associated with numerous vascular and thromboembolic complications. The mentioned flow conditions in the aorta during VA ECMO also lead to two essential phenomena, triggering further complications - increased systemic afterload and potentially differential hypoxemia or the so-called Harlequin syndrome [3–6]. The former one can result in an overload of the left ventricle, which, in turn, can lead to left ventricular dilation, increased left arterial pressure, and pulmonary edema, thus potentially causing further deterioration of heart function. In severe cases, the afterload is so high that the aortic valve no longer opens, favoring stasis in the left ventricle [3, 5]. The already damaged heart may not recover but instead, be further strained.

The latter complication is caused by the retrograde flow of oxygen-rich blood from the extracorporeal circuit encountering the anterograde flow of oxygen-poor blood, in the case of impaired lung function, from the left ventricle into the aorta. This can potentially prevent the supply of oxygen-rich blood to the coronary arteries and supra-aortic vessels [6]. The Harlequin syndrome is particularly critical if it leads to insufficient oxygen supply to the heart or brain. Consequently, the recovery of the damaged heart can be hindered, and neurological function can be permanently impaired [1].

These two issues in the use of VA ECMO are interacting phenomena. If the afterload is reduced, the risk of the Harlequin syndrome increases, and vice versa. The main influencing factors, outside of pulmonary function, are native heart contractility and ECMO flow rate since they determine the point in the aorta where blood from the heart and blood from the extracorporeal circuit mix [1].

Previous work on this topic has separately considered the phenomena of increased afterload and the Harlequin syndrome, individually, thereby posing a risk of addressing only one side of the problem. The issue of increased afterload has primarily been investigated using zero-dimensional simulation models of the cardiovascular system. This research found that as ECMO flow increases, afterload increases as well. It compared the use of intra-aortic balloon pump (IABP) and Impella (ECPELLA: combination of VA-ECMO and Impella) for reducing afterload [7–9]. The Harlequin syndrome has been studied with ultrasound [10, 11] and thermography measurements [12] in combination with circulatory mock loops. As expected, depending on heart function, varying ECMO flow rates are necessary to ensure oxygen supply to the heart and brain. Numerical studies employing three-dimensional computational fluid dynamics (CFD) simulations have also been used to further investigate aortic hemodynamics during VA ECMO. The studies ranged from CFD simulations in generic geometries [13] over patient-specific aortic arches [14–16] up to fluid-structure-interaction simulations [17]. All these studies did not consider the influence of various cannula locations on therapy outcome and did not evaluate the changes of afterload for the various support scenarios. Recent work by Wickramarachchi et al. [18] has addressed this question in an in-silico study, highlighting the influence of cannula tip orientation on oxygenation and afterload. However, combined mechanical circulatory support, especially with the Impella device has not been considered. Hence, there is no study which includes the influence of additional left ventricular unloading during ECPELLA and its effect on differential hypoxemia. Additionally, coronary arteries and perfusion of the heart were not included [18].

Finally, all existing CFD setups have modeled vessel oxygenation by including two fluids in the simulation, a fully oxygenated coming from the ECMO cannula and a non-oxygenated coming from the left ventricle. Oxygenation was then quantified by determining the fraction of blood coming from the VA ECMO. Such an approach has two important limitations. At first, one would expect high computational costs with such a multiphase model. Particularly modeling the mixing zone and properly describing the interface between the two fluids can be very challenging. Secondly, the assumption of a linear relationship between flow rate and oxygen saturation might be misleading, as it ignores the non-linearity of the oxygen dissociation curve.

Thus, within this study we introduce our computational model to investigate aortic hemodynamics during VA ECMO considering the additional use of the Impella device. We then use it to quantitatively assess the influence of different cannula tip locations and ECMO support scenarios on ventricular afterload and oxygen supply.

## Methods

### Geometry

A segmented computed tomography (CT) scan of a healthy female (age = 21 years) was used from the Vascular Model Repository (www.vascularmodel.com, model name 0028_H_ABAO_H) including the ascending aorta, the aortic arch, the thoracic and the abdominal aorta [19]. The geometry was cut distal to the iliac bifurcation and coronary arteries were additionally modelled using Materialise 3-matics (Materialise NV, Leuven, Belgium). In addition, an inlet section was added at the aortic sinus.

**Fig. 1:**
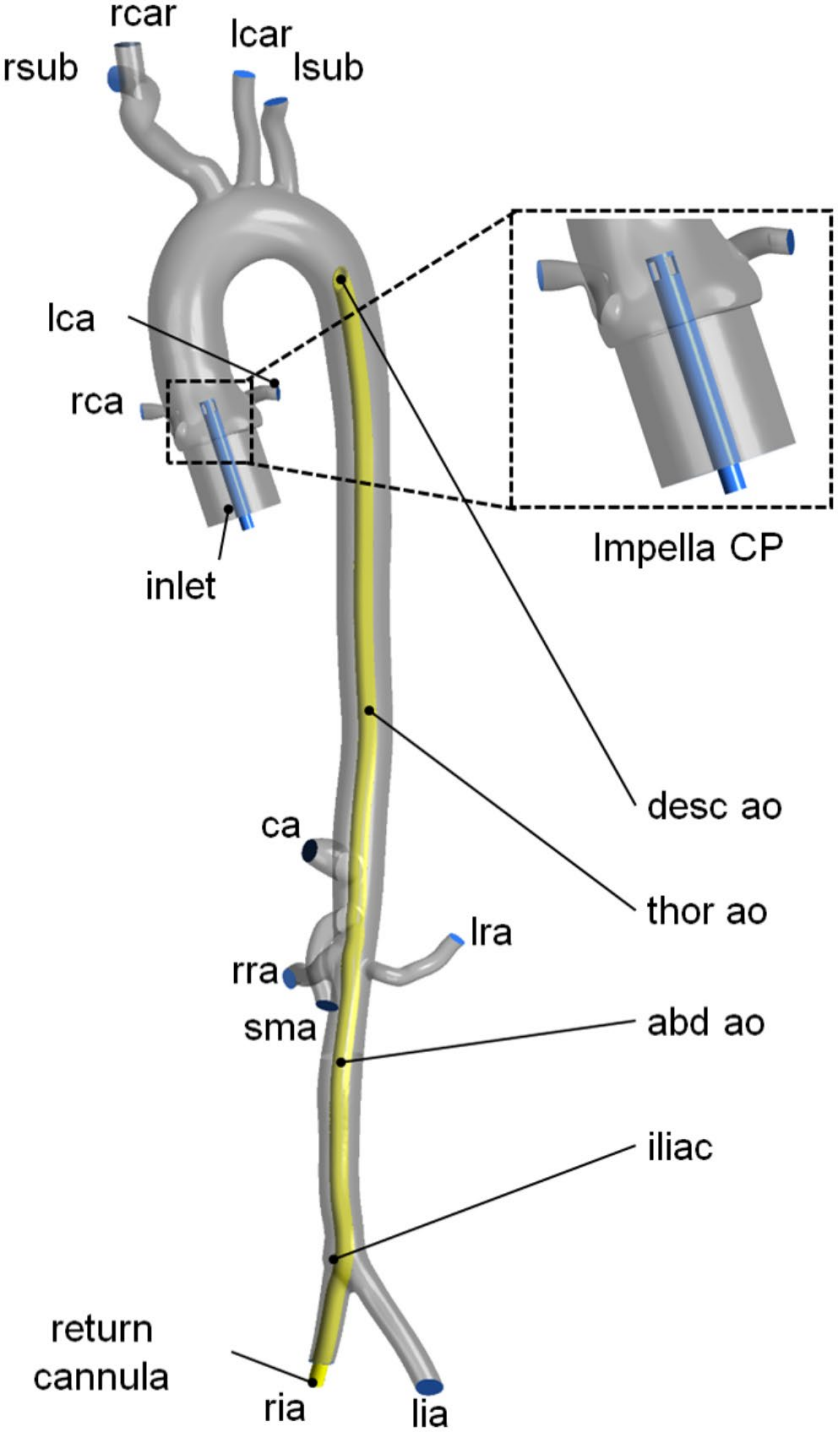
Vessel geometry, cannula tip locations, and close-up of the Impella CP device. Vessel names: rca - right coronary artery, lca - left coronary artery, rsub - right subclavian artery, rcar - right carotid artery, lcar - left carotid artery, lsub - left subclavian artery, ca. - celiac artery, sma - superior mesentric artery, rra - right renal artery, lra - left renal artery, ria - right iliac artery, lia - left iliac artery

A 17 Fr arterial cannula tip was positioned in four locations - iliac artery (*iliac*), abdominal aorta (*abd ao*), thoracic aorta (*thor ao*), and descending aorta (*desc ao*) such that retrograde perfusion is always occuring. Finally, an Impella CP pump was designed and added in the center of the CFD model inlet. The design was taken from available information online and comprised a representation of the pump’s outflow cannula. The model geometry, the cannula positions, the Impella CP device, and the vessel names are shown in Fig. 1. Only the most proximal cannula position in the descending aorta is visualized. It has to be noted that different cannulation sites such as central cannulation or axillary artery cannulation during VA ECMO exist in clinical practice [20]. Those would more likely be chosen over the rather uncommon tip locations *thor ao* and *desc ao*. However, the goal of the study was to systematically evaluate tip location in the aorta with an increasing distance to the aortic valve and its effect on organ perfusion and afterload. To facilitate a manageable number of scenarios, other cannulation locations were excluded.

### Simulation Setup

A CFD model was created with ANSYS Fluent 2022 R2 (Ansys Inc., Canonsburg, USA) using a Newtonian blood model with a density of 1054.6 kg/m^3 and viscosity of 3.6 mPas, and the k-omega SST turbulence model. Pulsatile inflow was prescribed at the inlet boundary and a constant inflow was prescribed at the cannula boundary. The individual scenarios and flow rates are explained in a paragraph below. All remaining boundaries were set as pressure outlets with a coupled three-element Windkessel model through a user defined function. In brief, in every time step of the CFD simulation the volume flow rates at the vessel boundaries were measured and provided as an input to the three-element Windkessel model. This model describes the relationship between flow and pressure through ordinary differential equations, that were solved with an implicit Euler approach for the pressure. Then, the pressure was passed back to the CFD simulation as the boundary condition for the next time step. A more detailed description of the multiscale coupling can be found in [21]. In addition, the final CFD model setup with the geometry and the user defined function for the Windkessel coupling can be found on https://zenodo.org/doi/10.5281/zenodo.10991265. The parameter values were taken from [13] and adjusted to achieve stability of the model. Most of the parameters are similar to the studies [13] and [18]. However, some changes had to be made as coronary arteries were not included in the studies mentioned above. All Windkessel parameters are shown in Table 1. Oxygen transport was modeled through a convectively transported user defined scalar of the variable partial pressure O_2_ (pO_2_). A value of 40mmHg was set at the native cardiac (LV) inlet representing pulmonary dysfunction and a value of 565 mmHg was set at the return cannula. The oxygen saturation (sO_2_) was then computed by taking the cycle averaged value of the pO_2_ concentration at the respective vessel boundary. Subsequently, the oxygen dissociation curve according to Eq. 1 [22] was used to map the partial pressures to the oxygen saturation in the range between 0 and 100%.

**Table 1:** Windkessel parameters R_p_: proximal resistance, C: compliance, R_d_: distal resistance. Vessel names: rca - right coronary artery, lca - left coronary artery, rsub - right subclavian artery, rcar - right carotid artery, lcar - left carotid artery, lsub - left subclavian artery, ca. - celiac artery, sma - superior mesentric artery, rra - right renal artery, lra - left renal artery, ria - right iliac artery, lia - left iliac artery

Boundary name	Rp [mmHg*s/ml]	C [ml/mmHg]	Rd [mmHg*s/ml]
rca	0.4	0.01	24
lca	0.4	0.01	24
rsub	1.56	0.014	15.91
rcar	1.56	0.029	15.91
lcar	2.87	0.01	39.16
lsub	1.48	0.047	9.76
ca.	0.64	0.44	5.68
sma	0.22	1.31	4.13
rra	0.23	2.56	4.04
lra	0.23	2.56	4.04
ria	0.48	0.11	7.63
lia	0.73	0.07	7.63

1 $$ s{O_2} = \frac{{{{\left( {\frac{{p{O_2}}}{{{p_{_{50}}}}}} \right)}^n}}}{{{{\left( {\frac{{p{O_2}}}{{{p_{_{50}}}}}} \right)}^n}}}$$

SO_2_ and pO_2_ are the oxygen saturation and partial pressure, respectively. The remaining parameters were set to p_50_ = 29.15 mmHg and *n* = 2.84. The visualization of the relationship between sO_2_ and pO_2_ is shown in Supplementary Fig. 1.

### Mesh and Solver Settings

An unstructured tetrahedral mesh with five prism layers and a local refinement at the cannula tip was created with ANSYS ICEM (Ansys Inc., Canonsburg, USA). Mesh and cycle independence considered the transient flow distribution and the cycle averaged pO_2_ content as control variables and changes below 2% were deemed acceptable. In the end, 10 cycles were necessary to reach an independent solution with meshes between 2.9 and 5.9 million elements, depending on the cannula location. A pressure-based solver with the SIMPLE pressure-velocity coupling algorithm, second order spatial discretization and a time step size of 1e-3 s was set. Convergence at each time step was assumed when the scaled residuals decreased below 10e-4. Simulations were performed with an Intel Xeon CPU (20 cores @ 2.4 GHz) and 64 GB RAM. The runtime for each scenario was around 400 core hours.

### Investigated Scenarios

**Fig. 2:**
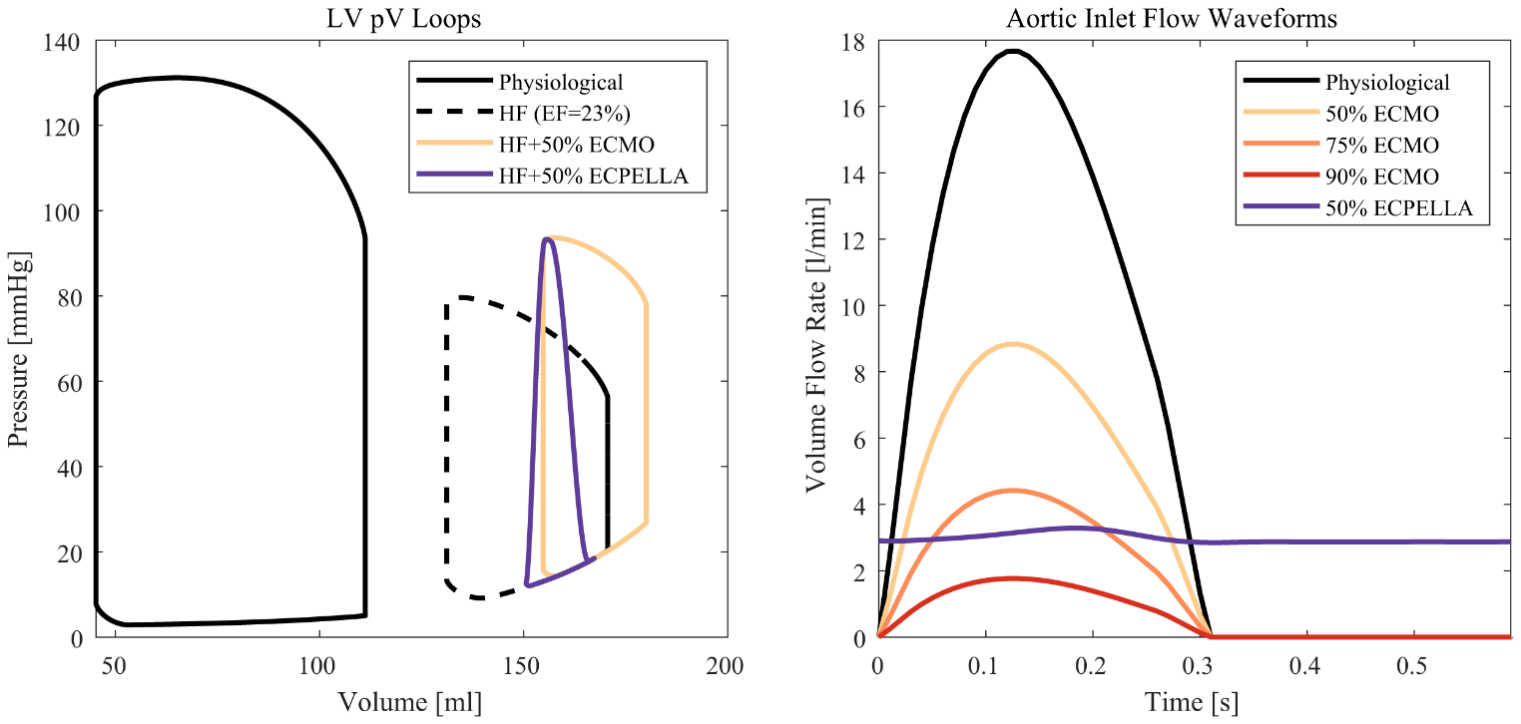
Left: Left ventricular pressure-volume loops in a physiological case, heart failure with 23% ejection fraction (HF), HF with 50% VA-ECMO support and HF with 50% ECPELLA support. Right: Inflow waveforms in l/min for the investigated scenarios

Three support scenarios (50%, 75% and 90% ECMO support) at four cannula locations - *iliac*, *abd ao*, *thor ao*, *desc ao* - with a total flow of 6 l/min were investigated. The volume flow curves at the inlet with an average value of 3 l/min, 1.5 l/min and 0.6 l/min are shown in Fig. 2 right. In addition, a control scenario (physiological) with a mean flow of 6/min and no ECMO flow was performed. Combined ECMO and Impella (50% ECPELLA) support was modeled at four cannula locations. To get information on the flow curves of the Impella pump, a lumped parameter model (LPM) from [21] was used. This model describes the blood flows and pressures of a cardiac failure patient under mechanical circulatory support (left ventricular assist device) and can be adjusted to specific pumps by implementing the individual HQ-curves of the devices. The model was expanded to include a VA-ECMO circulation and the Impella pump. As we did not have the specific HQ-curves of the Impella pump, a generic curve was assumed, such that a 50% ECPELLA support was established. After confirmation of reported behavior of left ventricular pressure-volume loops during cardiac failure, VA-ECMO and ECPELLA support [8], see Fig. 2 left, the flow curve of the Impella was extracted from the LPM and set in the CFD simulation (Fig. 2 right). At total, seventeen scenarios were investigated and oxygen saturation at the vessel boundaries, blood flow distribution in the geometry, and afterload changes were extracted. First, we evaluated the influence of tip locations and VA ECMO support scenarios (50%, 75%, 90%). Then, 50% ECPELLA was compared against 90% VA ECMO support. As no experimental data was available to validate the findings of the computational model, a comparison to existing studies in the literature has been performed. The computational studies of Wickramarachchi et al. [18], Stevens et al. [14], and Xi et al. [16] and the in-vitro study of Geier et al. [12] were used. Particularly, [18] has tackled a similar problem and allowed a detailed comparison of the results.

## Results

### Oxygen Saturation

**Fig. 3:**
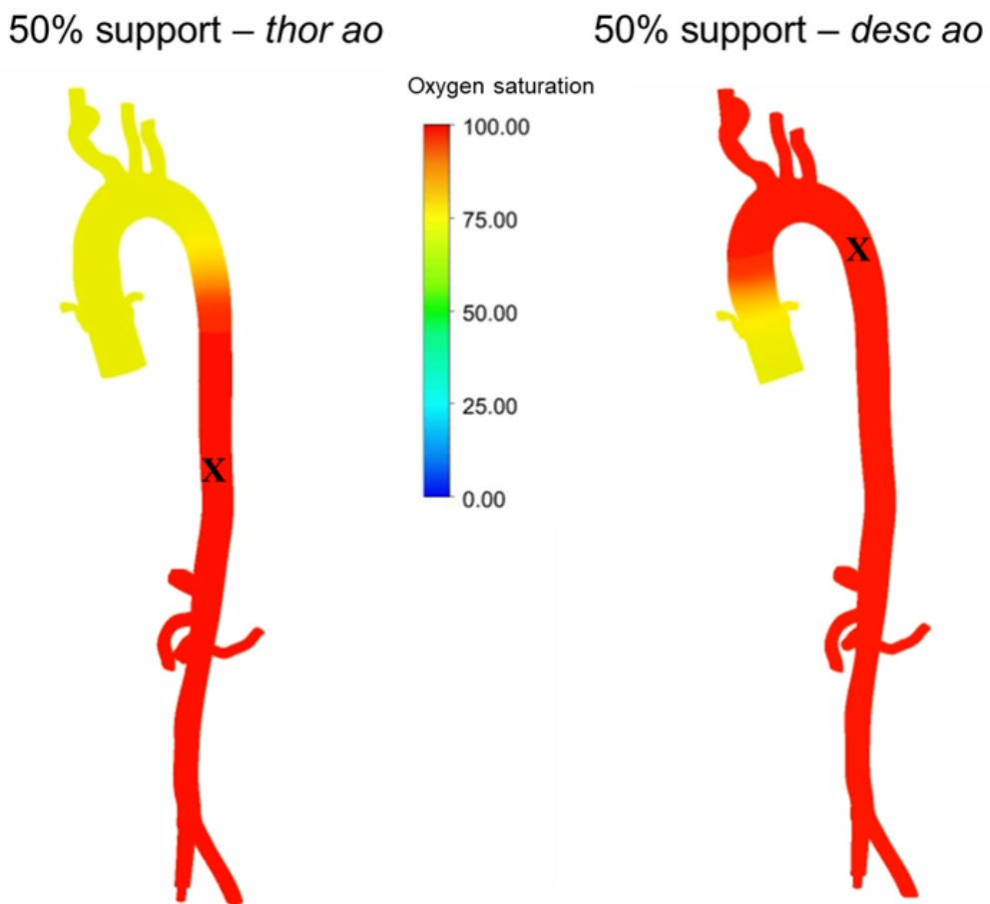
Oxygen saturation during *thor ao* and *desc ao* cannula tip location and 50% ECMO support. Cannula tip location is visualized with a black cross. The mixing zone during mid-diastole of the cardiac cycle is presented

Figure 3 shows the output of a CFD simulation with thoracic aorta cannula tip location and descending aorta cannula tip location during 50% ECMO support. A formation of a watershed region is observable which results in a lower oxygen saturation at the supra-aortic vessels during *thor ao* location. The videos of these two scenarios can be found in the Supplementary Material.

**Fig. 4:**
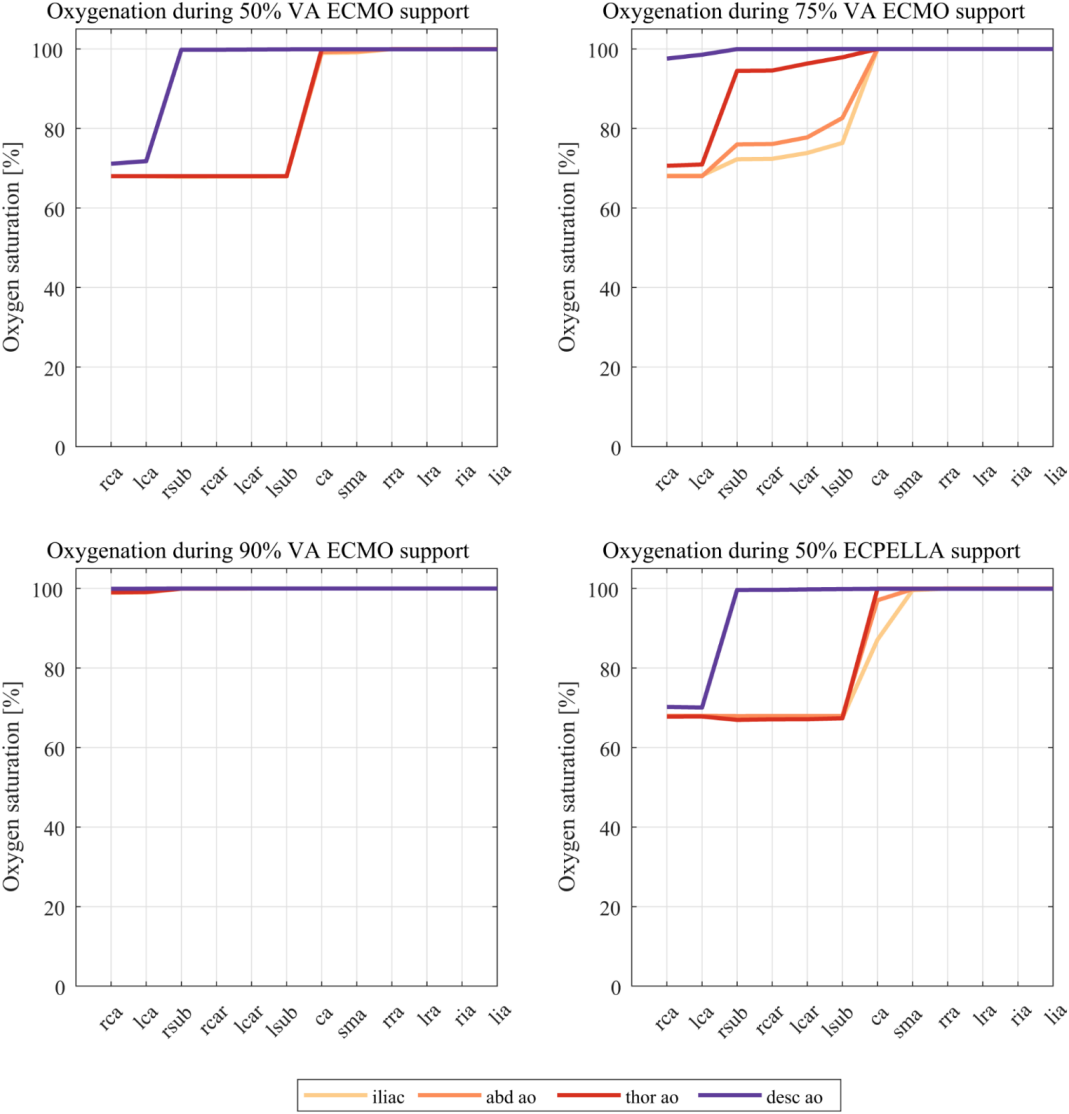
Oxygenation levels for all support scenarios and all cannula tip locations. Cycle-averaged values at each vessel boundary are shown. Vessel names: rca - right coronary artery, lca - left coronary artery, rsub - right subclavian artery, rcar - right carotid artery, lcar - left carotid artery, lsub - left subclavian artery, ca. - celiac artery, sma - superior mesentric artery, rra - right renal artery, lra - left renal artery, ria - right iliac artery, lia - left iliac artery

Cycle-averaged oxygen saturation for all support scenarios and cannula tip locations is shown in Fig. 4. The vessels are sorted from the most proximal to the most distal location in the vascular tree. Following observations can be made. During 50% VA ECMO support, the coronary arteries always experience reduced oxygen supply of approximately 70% independent of cannula tip location. The supra-aortic vessels are properly perfused during the most proximal (*desc ao*) tip orientation. For the remaining positions, the saturation is at 70%. During 75% ECMO support, *desc ao* location leads to proper oxygen supply to all vessels. The coronary arteries still receive low oxygenated blood during *thor ao*, *abd ao*, and *iliac* tip positions. Perfusion of the supra-aortic vessels is improved during more proximal tip location, with oxygen saturation levels between 75 and 95% percent. 90% VA ECMO support yields sufficient oxygen supply independent of cannula location. Shifting from 90% VA ECMO to 50% ECPELLA support yields a reduction of oxygen levels at the right and left coronary arteries, and supra-aortic vessels. The influence of cannula tip position is similar to the 50% VA ECMO case.

The distribution of oxygen partial pressures (pO_2_) to the individual vessels is shown in Supplementary Fig. 2. Although larger differences between the different cannula tip locations are seen, they are ultimately of lower relevance for the oxygen saturation that already reaches 99% at a value of 150 mmHg. The blood flow distribution to the vessels is shown in the Supplementary Material (Supplementary Fig. 3 and Supplementary Table 1). Only very small differences for various ECMO support scenarios and the different cannula tip locations were observed. There is no difference in blood flow distribution between native or Impella-supported LV output at 50% VA ECMO support. However, blood flow distribution during Impella-supported LV output and 90% VA ECMO support exhibits some differences. Coronary and supra-aortic vessel blood flow is about 10% lower in 50% ECPELLA support compared to 90% VA ECMO. Conversely, slightly more blood (5–10%) is transported to the lower body during 50% ECPELLA. Still, all results are in the range of physiological perfusion with a total cardiac output of 6 l/min.

### Afterload Changes

The cardiac afterload, defined as the pressure on the aortic valve (inlet), is presented in Table 2 with mean aortic pressures (MAP) for all conditions investigated. Afterload increases with increasing ECMO support reaching values up to 119 mmHg during 90% ECMO support and thoracic aorta location resulting in a 16% increase compared to the physiological scenario. Interestingly, the highest MAP values are not reached with the most proximal tip orientation but with the *thor ao* cannulation. ECPELLA support is able to decrease afterload compared to the 90% VA ECMO scenario by 8–17 mmHg (~ 8–14% reduction).

**Table 2:** Mean arterial pressures (MAP) for all support and cannula tip location conditions

Support condition	MAP [mmHg]			
	iliac	abd ao	thor ao	desc ao
50% ECMO	98	102	104	100
75% ECMO	101	103	112	104
90% ECMO	103	108	119	109
50% ECPELLA	95	100	102	98
Physiological	102			

**Fig. 5:**
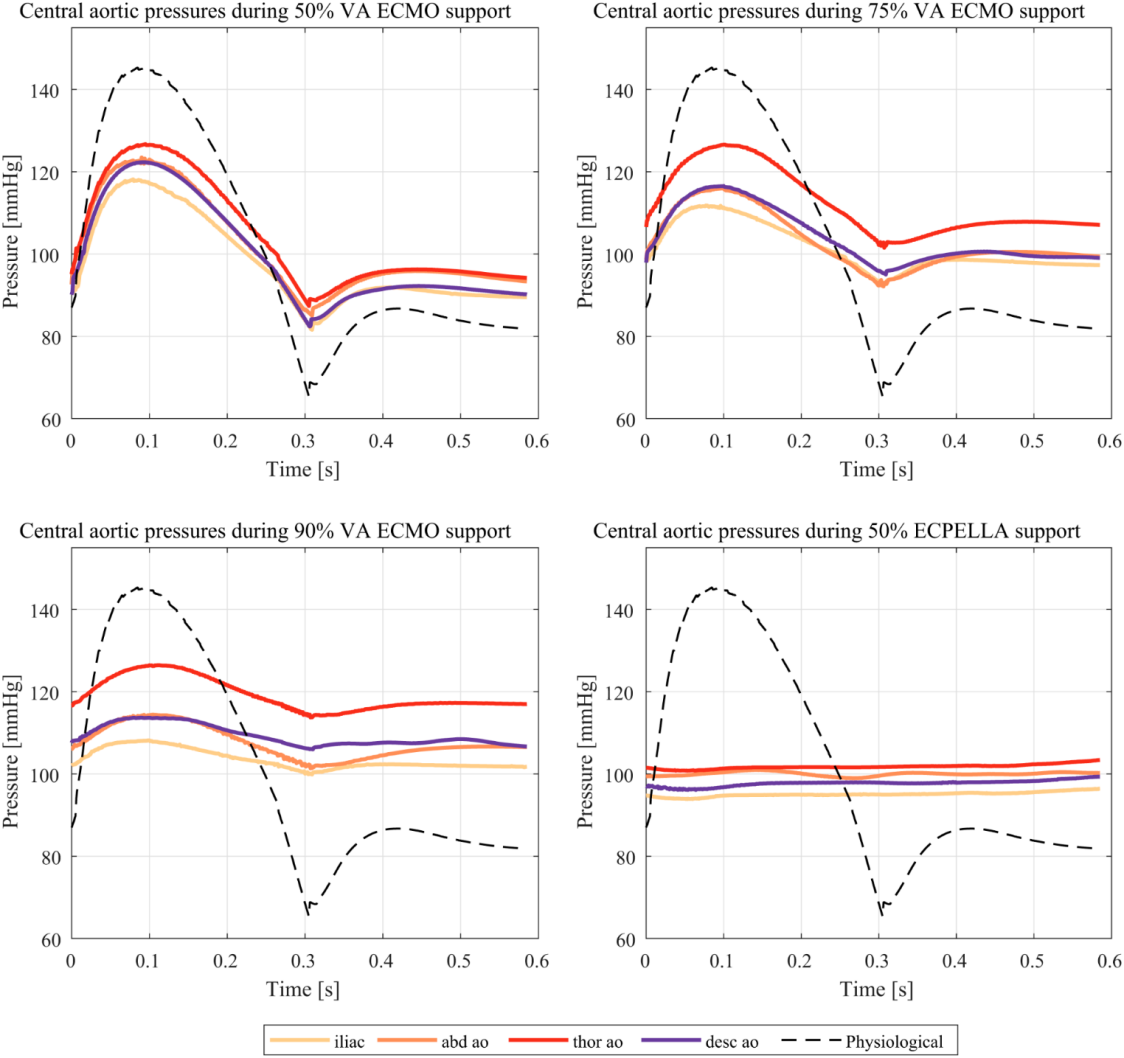
Central aortic pressures for all support scenarios and cannula tip locations. Results of the last cycle are shown

The transient central aortic pressures for all support scenarios and cannula tip locations are shown in Fig. 5. It can be see that the support scenario influences the pulse pressure with an increasing extracorporeal support resulting in lower pulsatility. Cannula location only marginally influences the pulsatility. For instance, 50% VA ECMO at *thor ao* has a pulse pressure of 39mmHg, 90% VA ECMO at *thor ao* has a pulse pressure of 13mmHg and 50% VA ECMO at *iliac* has a pulse pressure of 37mmHg. ECPELLA support results in almost constant pressures with small differences between the tip locations. A reduction of the maximal pressure during ECPELLA support of around 23 mmHg is observable. Finally, the highest pressures are always observed during *thor ao* and not the most proximal *desc ao* location as has already been noted in the analysis of the MAP (Table 2).

## Discussion

This study investigated aortic hemodynamics and oxygen distribution during VA ECMO support for three different support scenarios (50%, 75% and 90%) and four different cannula tip locations (iliac artery - *iliac*, abdominal aorta - *abd ao*, thoracic aorta - *thor ao*, descending aorta - *desc ao*). In addition, parallel use of the Impella pump (ECPELLA) in the 50% support scenario was simulated and compared to 90% VA ECMO support.

Analyzing all scenarios showed that a more proximal tip orientation is necessary to increase oxygen supply to the supra-aortic and coronary arteries for 50% and 75% support. During the 90% support scenario, proper oxygenation can be achieved independently of tip position. 50% ECPELLA support leads to a reduction of oxygen saturation at the coronary and supra-aortic arteries. There is no difference in vessel oxygenation at 50% VA ECMO whereby the remainder 50% of cardiac output is solely from native ejection or Impella-supported LV unloading. Systolic pressures are decreased by 20mmHg and cycle averaged pressures by 8–17 mmHg during the ECPELLA scenario. More proximal cannula location increases afterload. However, an unexpected decrease of pressure was found between *thor ao* and *desc ao*. We explain this finding by the rather complex flow physics and the interaction between the left ventricular jet and cannula jet. The study by Wickramarachchi et al. [18] has made the same observation, despite having used a different vascular geometry and simulation setup. It has to be noted that the absolute values MAPs during ECMO are typically lower between 65 and 80 mmHg. However, this difference is inconsequential to the presented results as only relative pressure changes to a baseline scenario were investigated. Further on, the same flow structures would develop with a lower baseline pressure level due to the rigid wall setting of the CFD model.

The remaining results of [18] compare to our findings in the following way. More proximal cannula tip location and higher VA ECMO support improved vessel oxygenation in [18]. In the 90% scenario they have identified sufficient oxygenation of all vessels except for the brachiocephalic artery. This vessel was only properly perfused during *desc ao*. In our case, we see proper oxygenation of all vessels, even the coronary arties with all cannula tip locations. This is an important difference, as the iliac artery is the most common cannula location during clinical use [2].

During the 80% VA ECMO scenario in [18], the brachiocephalic, the left common carotid artery and the left subclavian artery did not receive proper oxygen supply during *iliac, abd ao*, and *thor ao* cannula tip locations. This corresponds to our findings that show full oxygenation of all vessels during *desc ao* and 75% VA ECMO support and a reduced oxygenation at the cerebral and cardiac vessels for the remaining tip locations. At last, 50% VA ECMO support in [18] had insufficient oxygen supply up until the renal arteries (supra-aortic, celiac, and superior mesentric artery) for all locations. In our model, *desc ao* still delivers sufficient oxygen supply for all vessels except the coronary arteries. The remaining locations yield insufficient oxygen supply of the coronary and the supra-aortic arteries but other vessels are sufficiently perfused.

We attribute these differences to the way the oxygen supply was modeled. Wickramarachchi et al. have directly determined the vessel perfusion with blood coming from the VA ECMO and compared this value to physiological flow rates through the respective vessels. This assumes a linear relationship between the flow rate and the oxygen saturation, for instance, an increase from 0.5 to 1 l/min flow should also result in twice as high oxygen saturation. Such a strategy ignores the non-linear behavior of the oxygen dissociation curve as presented in Eq. 1.

In our simulations we explicitly model the transport of the oxygen partial pressure as a passive scalar and calculate oxygen saturation through the non-linear relationship in Eq. 1. This does not only allow mixing of blood coming from the left ventricle and the VA ECMO and thus a more realistic description of oxygen transport, but it also allows to consider further patient-specificity through modeling of the oxygen dissociation curve. It is well known, that parameters such as pH, temperature or hematocrit all influence the oxygen dissociation curve. Through changing of the curve parameters P_50_ and n (see Eq. 1), this behavior can also be described in the model.

Other studies in the literature allow a more qualitative comparison to our results. For instance, Xi et al. [16] have investigated the blood flow distribution in the aortic arch for different support scenarios. They did not observe large differences in the relative blood flow distribution, which is in correspondence with our results. The in-vitro study of Geier et al. [12] presents similar observations. At last, the computational study by Stevens et al. [14] has determined an extracorporeal support of at least 75% to achieve proper oxygenation of the supra-aortic vessels for a cannula tip location in the iliac artery. Once again, this corresponds with the observations made herein.

VA ECMO aims at proper oxygenation and simultaneously tries to achieve cardiac recovery which can be conflicting requirements, particularly when cardiac and brain tissue need to be sufficiently perfused with oxygenated blood from the extracorporeal circulation [23]. It is straightforward that higher ECMO flow and more proximal cannula tip orientation does increase oxygenation, however it also increases afterload. Naturally, a fine-tuning of the VA-ECMO is desirable. This includes a balance between between proper oxygenation and low extracorporeal support that does not overload the left ventricle. Our model can provide support for such choices. For instance, 75% VA ECMO support (4.5 l/min extracorporeal flow) and cannula tip location at the descending aorta performs as well as 90% VA ECMO support (5.4 l/min extracorporeal flow) at any cannula tip location in terms of vessel oxygenation. The additional use of the Impella pump can help to reduce afterload and provides an additional factor that influences the intricate interplay of proper oxygenation and ventricular overload. ECMO pumps are operated with constant rotational speeds and deliver blood flow with very low pulsatility. However, there are studies with devices (i-cor system of Xenios AG, Heilbronn, Germany) which allow a pulsatile pump operation that is synchronized with the cardiac cycle [24]. The effect of pulsatile flow on afterload and organ oxygenation considering the interplay of residual cardiac function and additional mechanical circulatory support systems is a very complex question that can be investigated with the model presented herein. Further VA ECMO cannulation locations [20] and the effect of retrograde, perpendicular or antegrade outflow can also be included and its effect evaluated.

### Limitations

Concerning the modeling strategy, the limitations are rigid wall assumptions and use of Windkessel parameters from healthy patients. A rigid wall seems to be an acceptable simplification, particularly when the focus of the model is on the oxygenation and not on the wall deformation and the wall shear stresses, as for instance in the study [13]. Further on, the work by Pons et al. [25] has compared aortic flow using CFD and fluid-structure-interaction (FSI) simulations with 4D Flow Magnetic Resonance Imaging (4D Flow MRI) measurements. Although there was a slight overestimation of the blood flow rates during CFD, a very good correlation between 4D Flow MRI and CFD was achieved. Another study of aortic flow during cardiopulmonary bypass confirms these observations [26]. Finally, computational costs of FSI simulations can be increased by a factor of 5–10 and many investigating all seventeen scenarios for 10 cardiac cycles might be prohibitive. Windkessel parameters might be different in VA ECMO patients and they are decisive for the distribution of the flow in the vascular tree. However, as there is not much information available on those values, we believe that it is better to keep them constant for a more consistent comparison of the different scenarios.

In addition, the pO_2_ at the return cannula of 565 mmHg is rather high and lower values < 450 mmHg are more common in clinical practice. As seen in Supplementary Fig. 2, full oxygen saturation is already reached at 150 mmHg and lowering the pO_2_ will most certainly not affect the overall results presented in this paper. Nevertheless, future studies could explore the influence of post-oxygenator oxygen partial pressure on vessel oxygenation. Further on, only a generic Impella device was modeled in the LPM to determine the inflow curves for the CFD simulations. As we had no information on the HQ-curve of the pump, the pump parameters in the LPM were adjusted until the behavior of the left ventricular pressure-volume loop corresponded to observations in clinic (see Fig. 2, left side). In this work, only one Impella support scenario (3 l/min and full cardiac support) was investigated. However, other cases can also be analyzed and more interaction between the native circulation and the various mechanical circulatory support systems can be considered. This can be achieved by closed-loop multiscale simulations with a bi-directional coupling of the aortic hemodynamics (CFD) and the hemodynamics of the cardiovascular system (LPM) as has been performed in [21] for ventricular assist device support.

Taken together, further work on the modeling side should consider (a) multiscale coupling between CFD and LPM and (b) further patient geometries to describe the influence of geometrical variations on the results.

Experimental validation, particularly using in-vivo blood flow visualization is imperative to move the theoretical studies further towards clinical application. As 4D Flow MRI is prohibitive in the context of ECMO, ultrasound techniques should be considered. Recent studies have shown the clinical feasibility of contrast-enhanced ultrasound to detect the watershed region in patients [27]. In addition, the study [11] has introduced ultrasound vector flow imaging to visualize aortic blood flow during VA ECMO and applied it in an in-vitro phantom setup.

## Conclusion

In this study, a computational model of aortic blood flow and oxygen transport during VA ECMO with and without combined Impella support was introduced. The influence of ECMO flow and cannula location on oxygenation and afterload changes was investigated. High support conditions (> 75% ECMO) and more proximal cannula locations are suggested to achieve proper oxygenation, particularly of brain and cardiac tissue. Afterload can be decreased through the use of the Impella by 8–17 mmHg, however oxygenation of coronary and supra-aortic arteries is decreased as well with values similar to 50% VA ECMO support. Our computational setup provides a more realistic description of oxygen transport than the existing models in the scientific literature as it includes the possibility to consider patient-specific oxygen binding into the simulations.

## Electronic Supplementary Material

Below is the link to the electronic supplementary material.

Supplementary Material 1

Supplementary Material 2

Supplementary Material 4

## Data Availability

The raw data can be retrieved by request from the authors. The CFD model is available at https://zenodo.org/doi/10.5281/zenodo.10991265.
