# Supplementary material for: Investigations of Differential Hypoxemia During Venoarterial Membrane Oxygenation with and Without Impella Support
Source: Cardiovasc Eng Technol. 2024 Jun 27;15(5):623–32. doi: 10.1007/s13239-024-00739-w (PMC11582155; doi:10.1007/s13239-024-00739-w)
Supplement: Supplementary file 3 — Supplementary Material 4 [file 13239_2024_739_MOESM4_ESM.docx]

**Investigations of differential hypoxemia during venoarterial membrane oxygenation with and without Impella support**

Michael Neidlin^1,*^, Ali Amiri^1^, Kristin Hugenroth^2^, Ulrich Steinseifer^1^

# Affiliations:

1: Department of Cardiovascular Engineering, Institute of Applied Medical Engineering, Medical Faculty, RWTH Aachen University, Aachen, Germany

2: enmodes GmbH, Aachen, Germany

# *Correspondence:

Name: Michael Neidlin

Address: Department of Cardiovascular Engineering, Institute of Applied Medical Engineering, Forckenbeckstr. 55

52074 Aachen, Germany

Email address: [neidlin@ame.rwth-aachen.de](mailto:neidlin@ame.rwth-aachen.de)


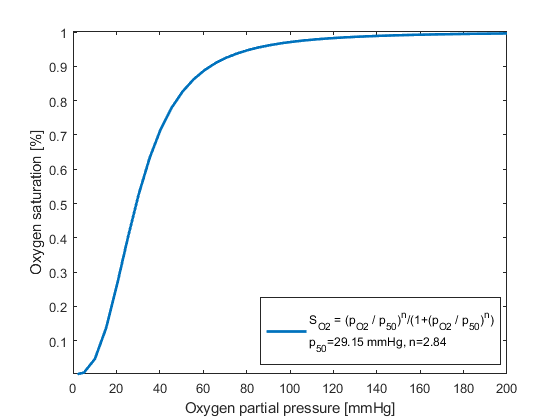


Supplementary Figure 1: The relationship between oxygen saturation and oxygen partial pressure for a range between 0-200 mmHg according to equation 1 from the main text.


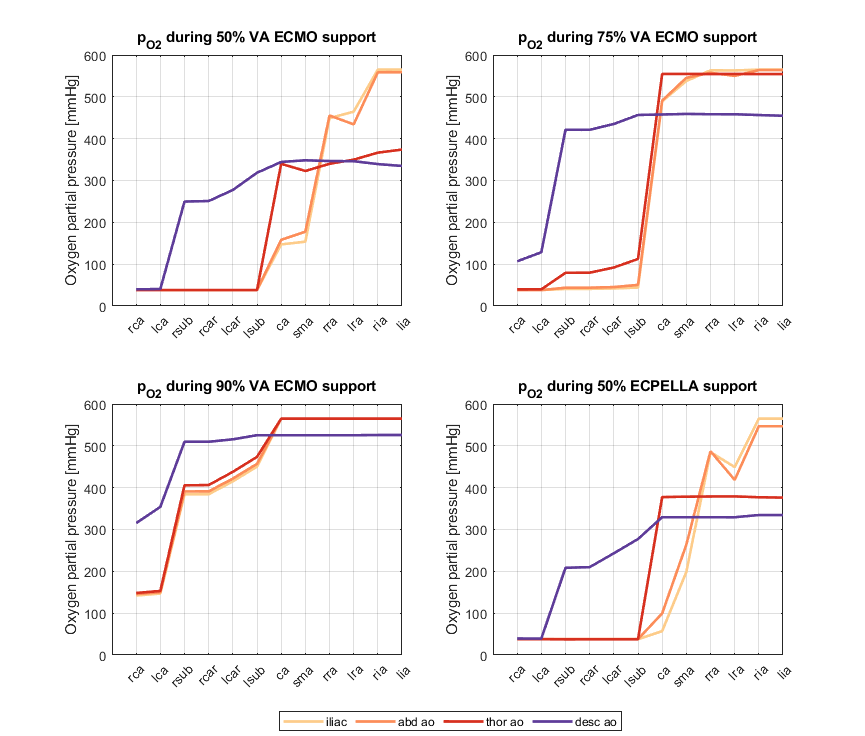


Supplementary Figure 2: Oxygen partial pressure at the different cannula tip positions and support scenarios. Vessel names: rca - right coronary artery, lca - left coronary artery, rsub - right subclavian artery, rcar - right carotid artery, lcar - left carotid artery, lsub - left subclavian artery, ca - celiac artery, sma - superior mesentric artery, rra - right renal artery, lra - left renal artery, ria - right iliac artery, lia - left iliac artery


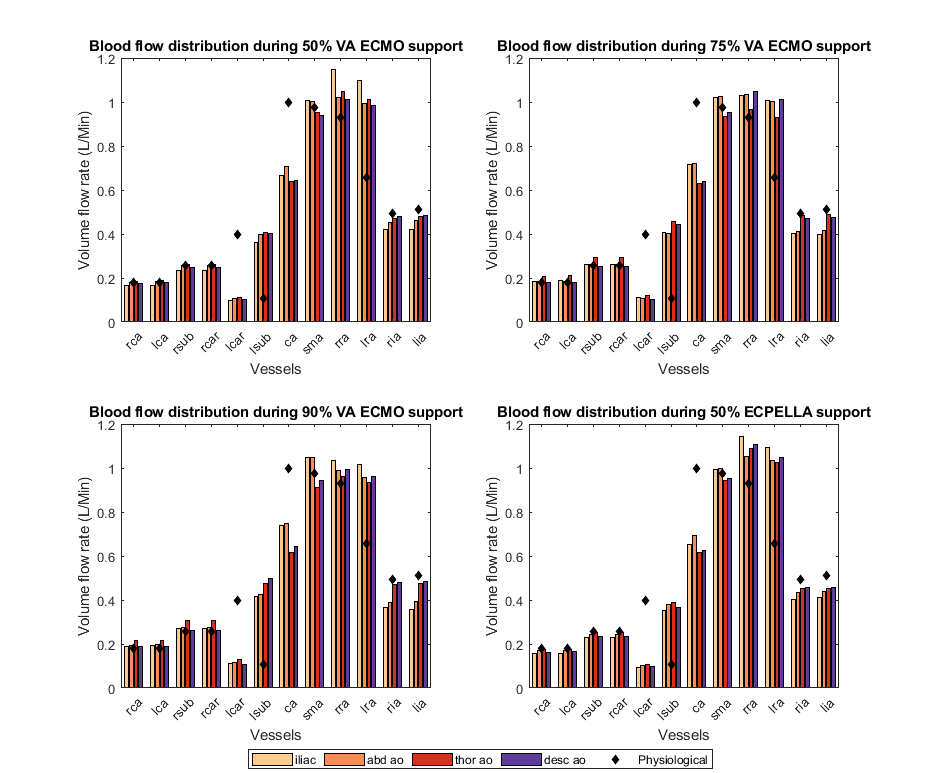


Supplementary Figure 3: Absolute blood flow distribution for all support scenarios and cannulation locations. Vessel names: rca - right coronary artery, lca - left coronary artery, rsub - right subclavian artery, rcar - right carotid artery, lcar - left carotid artery, lsub - left subclavian artery, rra - right renal artery, lra - left renal artery, sma - superior mesentric artery, ca - celiac artery, ria - right iliac artery, lia - left iliac artery

Supplementary Table 1: Relative blood flow distribution shown in mean and standard deviation (SD) for the different ECMO support scenarios. Vessel names: rca - right coronary artery, lca - left coronary artery, rsub - right subclavian artery, rcar - right carotid artery, lcar - left carotid artery, lsub - left subclavian artery, rra - right renal artery, lra - left renal artery, sma - superior mesentric artery, ca - celiac artery, ria - right iliac artery, lia - left iliac artery

|  | 50% VA ECMO | | 75% VA ECMO | | 90% VA ECMO | | 50% ECPELLA | |
| --- | --- | --- | --- | --- | --- | --- | --- | --- |
|  | mean | SD | mean | SD | mean | SD | mean | SD |
| rca | 2.9 | 0.1 | 3.1 | 0.2 | 3.3 | 0.2 | 2.8 | 0.1 |
| lca | 3 | 0.1 | 3.2 | 0.2 | 3.3 | 0.2 | 2.8 | 0.1 |
| rsub | 4.2 | 0.2 | 4.4 | 0.3 | 4.6 | 0.3 | 4 | 0.2 |
| rcar | 4.2 | 0.2 | 4.5 | 0.3 | 4.6 | 0.3 | 4.1 | 0.2 |
| lcar | 1.7 | 0.1 | 1.9 | 0.1 | 1.9 | 0.1 | 1.7 | 0.1 |
| lsub | 6.5 | 0.3 | 7.1 | 0.4 | 7.6 | 0.6 | 6.3 | 0.3 |
| ca | 11 | 0.5 | 11.3 | 0.8 | 11.4 | 1.1 | 10.9 | 0.6 |
| sma | 16.2 | 0.6 | 16.4 | 0.8 | 16.4 | 1.2 | 16.4 | 0.5 |
| rra | 17.6 | 1 | 17 | 0.6 | 16.5 | 0.5 | 18.6 | 0.6 |
| lra | 17 | 0.9 | 16.5 | 0.6 | 16.1 | 0.6 | 17.8 | 0.5 |
| ria | 7.6 | 0.4 | 7.4 | 0.7 | 7.1 | 0.9 | 7.4 | 0.4 |
| lia | 7.7 | 0.5 | 7.4 | 0.7 | 7.1 | 1 | 7.4 | 0.3 |
